# The use of procalcitonin as an antimicrobial stewardship tool and a predictor of disease severity in coronavirus disease 2019 (COVID-19)

**DOI:** 10.1017/ice.2021.28

**Published:** 2021-01-25

**Authors:** George P. Drewett, Olivia C. Smibert, Natasha E. Holmes, Jason A. Trubiano

**Affiliations:** 1 Department of Infectious Diseases, Austin Health, Heidelberg, Australia; 2 Department of Infectious Diseases, Peter MacCallum Cancer Centre, Melbourne, Australia; 3 Department of Oncology, Peter MacCallum Cancer Centre, University of Melbourne, Parkville, Australia; 4 Department of Critical Care, University of Melbourne, Parkville, Australia; 5 Data Analytics Research and Evaluation (DARE) Centre, Austin Health and University of Melbourne, Heidelberg, Australia; 6 Department of Medicine, University of Melbourne, Heidelberg, Australia

*To the Editor*—Prior to the coronavirus disease 2019 (COVID-19) pandemic, serum procalcitonin (PCT)-based antimicrobial stewardship (AMS) algorithms have been shown to be effective at differentiating between bacterial and nonbacterial respiratory tract infection, leading to improved mortality, less antibiotic use, and decreased risk of antibiotic side effects.^1^ In the context of COVID-19, initial reports of the utility of PCT were from hospitalized patients in whom PCT was found to correlate with disease severity, longer intensive care unit (ICU) stay, and inpatient mortality, along with a range of other biochemical markers.^2–4^ However, PCT would have unique value if its measurement at admission for COVID-19 or at time of clinical deterioration could be an important discriminator between bacterial coinfection versus noninfectious cause, allowing for improved AMS.^7^ Widespread use of empiric antibiotics is occurring globally in patients hospitalized with COVID-19, despite low rates of microbiologically proven bacterial infection.^5,6^

We investigated whether PCT was associated with commencement of antibiotic therapy. Additionally, we examined whether PCT was associated with duration of antibiotic therapy, intravenous-to-oral antibiotic switch, and other clinical and biochemical markers of COVID-19 severity.

A single-center, prospective, observational cohort study of patients with COVID-19 admitted to Austin Health (Melbourne, Australia) was undertaken. All patients were admitted to a specialized, multidisciplinary unit coordinated by infectious diseases physicians. PCT was measured at the time of ICU admission or at clinician discretion outside the ICU. Patients were stratified based on their initial PCT measurement into normal (<0.07 µg/L), medium (0.07 µg/L – 0.5 µg/L) and high (>0.5 µg/L) groups, based on consensus guidelines and recently published data in a COVID-19 patient group.^5,8^ Demographic, clinical, and laboratory data were also collected. Statistical analysis was performed using Stata version MP 16.1 software (StataCorp, College Station, TX). We used χ^2^ and rank-sum tests for univariate analysis according to PCT strata. Given the limited sample size, multivariable analysis was not performed.

In total, 166 patients were admitted with COVID-19 at the Austin Hospital between March and September 2020. Of these, 55 had at least 1 PCT measurement during their admission (Table 1). Most patients had PCT measured within the first day of admission (median days, 1; interquartile range [IQR], 0–3). Blood cultures were taken in 42 of 55 patients, with 3 positive results (4.7%) (Blood culture organisms: *Enterobacter cloacae*, PCT 38.8 µg/L; *Escherichia coli*, PCT 1.37 µg/L; *Staphylococcus epidermidis*, PCT 0.14 µg/L).

**Table 1.** Demographics, Laboratory Parameters, Treatment Parameters, and Outcomes for Normal PCT, Medium PCT, and High PCT Groups

Variable	Normal PCT Group (PCT <0.07 µg/L)	Medium PCT Group (PCT 0.07–0.5 µg/L)	High PCT Group (PCT >0.5 µg/L)	*P* Value
Number	15	27	13	
**Demographics**				
Age median y (IQR)	61 (34–82) (n = 15)	66 (59–76) (n = 27)	62 (56–79) (n = 13)	.81
Sex, female, no. (%)	9 (60)	11 (41)	7 (54)	.45
Charlson comorbidity index (age adjusted), median (IQR)	4 (0–6) (n = 15)	3 (2–5) (n = 26)	5 (2–7) (n = 11)	.64
**Laboratory parameters, median (IQR)**				
CRP (mg/L)	23.1 (18.3–40.6) (n = 15)	117 (41.4–193) (n = 25)	172 (110–204) (n = 13)	**<.01**
Hemoglobin (g/L)	135 (116–148) (n = 15)	139 (121–142) (n = 27)	121 (111–133) (n = 13)	.27
White blood cell count ×10^9^/L	5.2 (4.3–7.1) (n = 15)	6.2 (4.3–8) (n = 27)	6.4 (4.2–8.8) (n = 13)	.38
Lymphocytes ×10^9^/L	1 (0.8–1.5) (n = 15)	0.8 (0.5–1) (n = 27)	0.7 (0.6–0.8) (n = 13)	**<.01**
D-dimer (ng/mL)	599 (395–972) (n = 15)	969 (503–1,386) (n = 25)	1,215 (832–1,705) (n = 13)	.22
Ferritin (mcg/mL)	230 (126–448) (n = 14)	1084 (439–1551) (n = 25)	525 (408–631) (n = 13)	**<.01**
LDH (units/L)	262 (206–269) (n = 9)	376.5 (337–496.5) (n = 16)	330 (280–525) (n = 10)	**.01**
**Treatment parameters, no. (%)**				
Receipt of antibiotics	9 (60)	22 (81)	13 (100)	**.03**
Remdesivir	3 (20)	9 (33)	4 (31)	.65
Dexamethasone	6 (40)	18 (67)	9 (69)	.18
**Outcomes, no. (%)**				
Admission to ICU	3 (20)	13 (48)	6 (46)	.18
Requirement for >24 h supplemental O_2_	5 (33)	21 (78)	13 (100)	**<.01**
Mechanical ventilation	0 (0)	7 (26)	2 (15)	.09
Duration of antibiotics (IV+PO), median d (IQR)	4 (3–5) (n = 9)	4 (2–6) (n = 23)	4.5 (4–8.5) (n = 12)	.50
Time to oral stepdown, median d (IQR)	2 (1–3) (n = 5)	2 (1–2) (n = 13)	3.5 (2.5–4.5) (n = 8)	**.04**

Note. PCT, serum procalcitonin; CRP, C-reactive protein; LDH, lactate dehydrogenase; IQR, interquartile range; O_2_, oxygen; IV, intravenous; PO, oral; ICU, intensive care unit.

PCT levels were significantly associated with antibiotic use (*P* = .03). In total, 44 of 55 patients (80%) received antibiotic therapy during their admission. Of 15 patients, 9 (60%) with normal PCT received antibiotic therapy during their admission, compared with 22 of 27 patients (81%) in the medium PCT group, and 13 of 13 patients (100%) in the high PCT group. In those who received antibiotics, PCT was not associated with total duration of antibiotic therapy (*P* = .50). PCT was associated with earlier de-escalation to oral therapy, with a median duration of 2 days prior to step down in the normal and medium PCT groups versus 3.5 days in the high PCT group (*P* = .04).

PCT levels were associated with supplemental oxygen requirements during admission. Moreover, 100% of patients in the high PCT group required supplemental oxygen, compared with only 35% of patients in the normal PCT group (*P* < .01). PCT was not associated with requirement for ICU admission or with dexamethasone and remdesivir therapy. Serum PCT levels were associated with C-reactive protein (CRP) (*P* < .01), lymphocytes (*P ≤* .01), ferritin (*P* < .01), and lactate dehydrogenase (LDH) (*P* = .01).

In our experience with PCT in COVID-19, we have noted that changes in serum PCT are associated with both initiation of antibiotic therapy, and intravenous-to-oral switch. These findings highlight the potential utility of PCT as a component of antimicrobial stewardship interventions.^10,11^ Indeed, all patients in the high PCT group received antibiotics during their admissions, while 20% in the medium PCT group and 40% in the low PCT group did not receive any antibiotic therapy. This finding suggests that clinicians were more comfortable withholding antibiotic therapy in patients with lower PCT, which may represent a stewardship intervention opportunity.

Once initiated, the total duration of antibiotic therapy (intravenous [IV] and oral) was the same across the groups. However, we found a significant association between PCT and de-escalation to oral therapy. Patients in the high PCT group received, on average, 1.5 days of additional IV antibiotics compared to those in the medium- and low-PCT groups, suggesting increased clinician comfort in de-escalating antibiotics for patients without high PCT.

Although our study was limited by small study size and nonrandomized design, our results still suggest that, in COVID-19 patients, measurement of PCT, in conjunction with other clinical assessment, may have a role in prognostication and decision-making algorithms for a wider group of patients than only those admitted to ICU, aiding AMS interventions in this cohort.

## References

[1] Schuetz P , Wirz Y , Sager R , et al. Procalcitonin to initiate or discontinue antibiotics in acute respiratory tract infections. Cochrane Database Syst Rev 2017;10:CD007498. 29025194 10.1002/14651858.CD007498.pub3 PMC6485408

[2] Chen G , Wu D , Guo W , et al. Clinical and immunological features of severe and moderate coronavirus disease 2019. J Clin Invest 2020;130:2620–2629. 32217835 10.1172/JCI137244 PMC7190990

[3] Heesom L , Rehnberg L , Nasim-Mohi M , et al. Procalcitonin as an antibiotic stewardship tool in COVID-19 patients in the intensive care unit. J Glob Antimicrob Resist 2020;22:782–784. 32717489 10.1016/j.jgar.2020.07.017 PMC7381395

[4] Chen T , Wu D , Chen H , et al. Clinical characteristics of 113 deceased patients with coronavirus disease 2019: retrospective study. BMJ 2020;368:m1091. 32217556 10.1136/bmj.m1091 PMC7190011

[5] Vaughn VM , Gandhi T , Petty LA , et al. Empiric antibacterial therapy and community-onset bacterial co-infection in patients hospitalized with COVID-19: a multi-hospital cohort study. Clin Infect Dis 2020. doi: 10.1093/cid/ciaa1239. PMC7499526 32820807

[6] Huttner BD , Catho G , Pano-Pardo JR , Pulcini C , Schouten J. COVID-19: don’t neglect antimicrobial stewardship principles! Clin Microbiol Infect 2020;26:808–810. 32360446 10.1016/j.cmi.2020.04.024 PMC7190532

[7] Han J , Gatheral T , Williams C. Procalcitonin for patient stratification and identification of bacterial coinfection in COVID-19. Clin Med (Lond) 2020;20(3):e47. 32414743 10.7861/clinmed.Let.20.3.3 PMC7354055

[8] Schuetz P , Beishuizen A , Broyles M , et al. Procalcitonin (PCT)-guided antibiotic stewardship: an international experts consensus on optimized clinical use. Clin Chem Lab Med 2019;57:1308–1318. 30721141 10.1515/cclm-2018-1181

[9] Harris PA , Taylor R , Thielke R , Payne J , Gonzalez N , Conde JG. Research electronic data capture (REDCap)—a metadata-driven methodology and workflow process for providing translational research informatics support. J Biomed Inform 2009;42:377–381. 18929686 10.1016/j.jbi.2008.08.010 PMC2700030

[10] Bennouar S , Bachir Cherif A , Kessira A , et al. Usefulness of biological markers in the early prediction of corona virus disease-2019 severity. Scand J Clin Lab Invest 2020;80:611–618. 32945705 10.1080/00365513.2020.1821396

[11] Sheng L , Wang X , Tang N , Meng F , Huang L , Li D. Clinical characteristics of moderate and severe cases with COVID-19 in Wuhan, China: a retrospective study. Clin Exp Med 2020. doi: 10.1007/s10238-020-00662-z. PMC7501760 32949308

[12] Team C-NIRS. COVID-19, Australia: epidemiology report 24 (fortnightly reporting period ending 30 August 2020). Commun Dis Intell 2020;44. 10.33321/cdi.2020.44.75 32907528

